# Novel Self-Dyed Wholly Aromatic Polyamide-Hydrazides Covalently Bonded with Azo Groups in Their Main Chains: 1. Structure-Property Relationships

**DOI:** 10.3390/molecules171213969

**Published:** 2012-11-26

**Authors:** Nadia A. Mohamed, Mohammad H. Sammour, Ali M. Elshafai

**Affiliations:** 1 Department of Chemistry, Faculty of Science, Cairo University, Giza 12613, Egypt; 2 Excellence Center of Science and Technology, Elsalam-2, Cairo 3066, Egypt; E-Mails: mhsamour@yahoo.com (M.H.S.); alielshafaie1@yahoo.com (A.M.E.)

**Keywords:** azopolyamide-hydrazides, intrinsic viscosity, solubility, crystallinity, mechanical properties, thermal characteristics

## Abstract

Twelve novel intrinsically colored wholly aromatic azopolyamide-hydrazides containing various proportions of *para*- and *meta*-phenylene units were successfully synthesized by a low temperature (−10 °C) solution polycondensation reaction of either 4-amino-3-hydroxybenzhydrazide (4A3HBH) or 3-amino-4-hydroxybenzhydrazide (3A4HBH) with an equimolar amount of either 4,4′-azodibenzoyl chloride (4,4′ADBC), 3,3′-azodibenzoyl chloride (3,3′ADBC), or mixtures of various molar ratios of 4,4′ADBC and 3,3′ADBC in anhydrous *N*,*N*-dimethyl acetamide (DMAc) containing 3% (wt/v) LiCl as a solvent. The structures of the polymers were proven by elemental analysis, FTIR, ^1^H- and ^13^C-NMR spectroscopy. The polymers’ properties were strongly affected by their various structures. The intrinsic viscosities of the polymers were ranged from 0.7 to 4.75 dL g^−1^ and increased with the *para*-phenylene units content. The polymers are partially soluble in DMAc, dimethyl formamide (DMF) and *N*-methyl-2-pyrrolidone (NMP). Their solubility increases with the introduction of *meta*-phenylene moieties into the polymer chains. The polymers exhibit a great affinity for water sorption. Their hydrophilicity increases as a function of the content of *meta*-phenylene rings incorporated into the polymer. Mechanical properties of the polymer films are improved markedly by substitution of *para*-phenylene units for *meta*-phenylene units. The completely *para*-oriented type polymer has the best thermal and thermo-oxidative stability relative to those of the other polymers.

## 1. Introduction

Polymeric products are usually dyed with a reactive dye or a disperse dye at a high temperature for a long time, which will waste energy and time. The use of dispersed pigments for coloring polymeric products gives rise to some problems such as limited attainable shades, nucleating effect, equipment cleaning, compatibility with polymeric products, wash fastness, migration, and so on. Thus, the use of intrinsically colored polymers, which have a chromophoric system (aromatic azo groups) forming part of their main chains, offers lots of advantages. In addition, the aromatic azo group is of special interest because of the existence of *cis-trans* isomerism and its effect on the photochromic and photocontractile properties of the polymers [1,2,3]. Further, the azo-*para*-phenylene moiety in the polymer main chain can impart rigidity to the polymer [4]. Therefore, polymers that contain the azo group have potential use in a variety of applications, such as materials with liquid-crystal [5,6], or non-linear optic properties [7]. Azobenzene-containing materials are photochromic and reversibly switch between two spectroscopically distinct forms by use of light. Thus azobenzene-based polymers are interesting due to their photoresponsive behavior which can be utilized in optical switches, optical data recording or optical information storage [8,9,10].

Wholly aromatic polyamide-hydrazides are a unique class of materials which combine the properties of both the aromatic polyamides and the aromatic polyhydrazides [11,12,13,14,15]. These polymers could be classified as high performance materials because they possess some interesting and potentially useful properties. They exhibit favorable rheological behavior that allowed for successful preparation of fibers and films from their solutions in dimethyl acetamide [16] and dimethyl sulfoxide [13]. Their appropriately drawn fibers showed very high mechanical strength and moduli, which are superior to that of glass fiber and steel wire [13]. These fibers can be successfully applied in fabrics, rigid composites, tire-cords, impact-absorbing devices, and for ballistic protection [17]. Polyamide-hydrazide films are useful as reverse osmosis membranes in water-pollution control, and for concentration of fruit juices [18], and as semipermeable membranes for desalination of artificial and natural seawater [19,20,21]. Further, their modified transition metal chelates have shown good electrical conductivity [22]. Moreover, these polymers are characterized by high thermal and thermo-oxidative stability [23,24]. Aromatic polyamide-hydrazides are well known as precursors to polyamide-1,3,4-oxadiazoles, which are one of the most important classes of chemically and thermally stable heterocyclic polymers [25,26]. It is well established that all aromatic polymers containing only benzoxazole [27,28] or 1,3,4-oxadiazole [29,30] moieties as the main structural feature of the polymer chains exhibit excellent thermal stability and other attractive physical properties.

Thus, our goal will be the synthesis and characterization of several novel self-colored wholly aromatic azopolyamide-hydrazides with *o*-hydroxyl groups which would be expected to undergo thermo-chemical transformation into linear aromatic polymers with alternating 1,3,4-oxadiazole and benzoxazole structural units within the same polymer backbone in order to classify these materials as a category of heat resistant polymers. 4-Amino-3-hydroxybenzhydrazide (4A3HBH), 3-amino-4-hydroxybenzhydrazide (3A4HBH), 4,4′-azodibenzoyl chloride (4,4′ADBC) and 3,3′-azodibenzoyl chloride (3,3′ADBC) were selected as monomers to be used in this study. The second phase of this work will be extended to study the effect of introducing different predetermined proportions of *para*- and *meta*-oriented phenylene moieties into the backbone chain of the polymers on their properties.

## 2. Results and Discussion

### 2.1. Polymer Synthesis

**Scheme 1:**
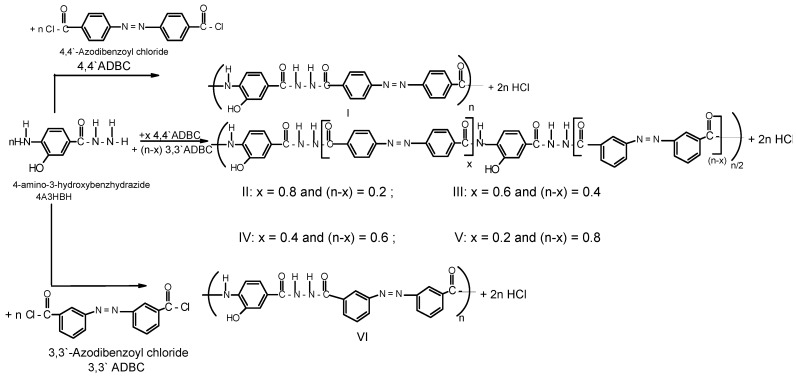
Typical synthetic scheme for novel wholly aromatic azopolyamide-hydrazides **I**-**VI**.

**Scheme 2:**
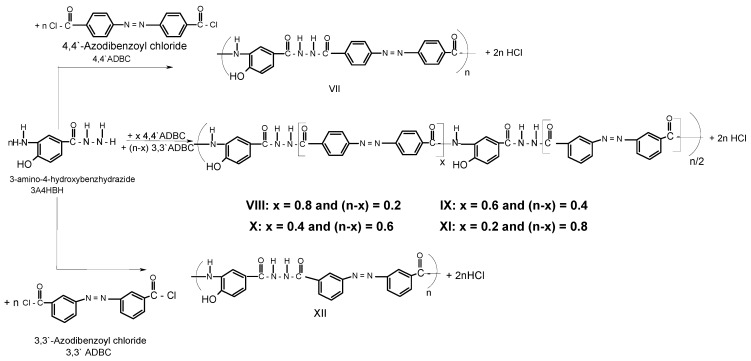
Typical synthetic scheme for novel wholly aromatic azopolyamide-hydrazides **VII**-**XII**.

A series of novel wholly aromatic azopolyamide-hydrazides has been synthesized by a low temperature (at –10 °C) solution [in anhydrous DMA_C_ containing 3% (wt/v) LiCl] polycondensation reaction of either 4A3HBH or 3A4HBH with stoichiometric amounts of either 4,4′ADBC, 3,3′ADBC or mixtures of various molar ratios of 4,4′ADBC, and 3,3′ADBC as summarized in Table 1 and illustrated in Scheme 1 and Scheme 2. The content of *para*- and *meta*-phenylene moieties was varied within this series so that the changes in the latter were 10 mole % from polymer to polymer, starting from an overall content of 0–100 mol %. Thus, all the polymers are structurally similar except for the mode of linking of phenylene groups in the polymer chain. All the polymers were produced in a quantitative yield which ranged between 99.38% and 99.95% (Table 1). The basic reaction employed here is the Schotten-Baumann condensation of an aromatic acid chloride and an aminohydrazide, which is generally known to be fast and quantitative. It is established that the rate by which hydrazide groups of aminobenzhydrazide reacted with acid chloride is about seven times greater than that of the amino groups with the same acid chloride [31,32]. These properties indicate that when this polycondensation is performed by gradual addition of solid acid chloride in a solution of aminobenzhydrazide monomer, the hydrazide groups of the latter react first, and the so-called “wholly ordered” polymer with alternating amide and hydrazide linkages is formed.

The liberation of HCl by-product has multifunctional effects on this reaction. First, HCl acts as a reaction catalyst. Second, it reacts with the solvent (DMAc) to form a salt (DMAc-HCl) [33]. Although, it could be expected that there is a competition between the growing polymer molecules and the solvent (DMAc) for HCl, solvent is more basic than the polymeric product, thus associating with solvent carbonyl oxygen predominates. This salt is useful in making and keeping in solution azopolyamide-hydrazides through enhancing the solubility of the growing polymer, thereby assisting in molecular weight build up and should direct the polycondensation equilibrium towards completion and be an additional driving force for the process. Consequently, since the basic reaction is known as rapid even at low temperatures, and, since one of the polycondensation products is continuously being withdrawn from the equilibrium, this condensation polymerization reaction is expected to proceed to acceptably high conversions and thus satisfy one of the mentioned requirements for preparation of high molecular weight products imposed by the nature of this step-growth process. Addition of LiCl to the polymerization medium enhances the solubility of the polymers. Although there is a competition between the polymer and solvent (DMAc) for the Li^+^ cation; the solvent is more basic than the polymeric product. Binding by the solvent predominates and the Li^+^ cation associates with the solvent carbonyl oxygen; this is useful for making and retaining the polymer in solution [34].

### 2.2. Polymer Identification

The structure of the prepared polymers is proposed on the basis of their elemental analyses, FTIR, ^1^H- and ^13^C-NMR spectra. The elemental analysis values are in good agreement with the theoretical ones calculated for the expected repeating unit (C_21_H_15_N_5_O_4_) as shown in Table 2. On the other hand, all the FTIR spectra of these polymers showed common absorption stretching vibration bands at the following wave numbers: 3650–3150 cm^−1^ (intense and broad) assigned to the overlapped =NH, -OH (phenolic) and the possible interchain hydrogen bonding; 2300–2180 cm^−1^ (weak) is attributed to possible enol-type configuration of hydrazide and amide groups; 1660–1650 cm^−1^ (strong) corresponded to the amide carbonyl group; 1580–1600 cm^−1^ indicated the aromatic carbon-carbon double bonds; 1525–1515 cm^−1^ is due to =NH of amide II; 1500 cm^−1^ indicated carbon-carbon single bond in ring; 1420 cm^−1^ is attributed to carbon-oxygen (phenolic); 1340, 1280 and 1250 cm^−1^ corresponded to carbon-hydrogen combined with =NH of amide III.

**Figure 1:**
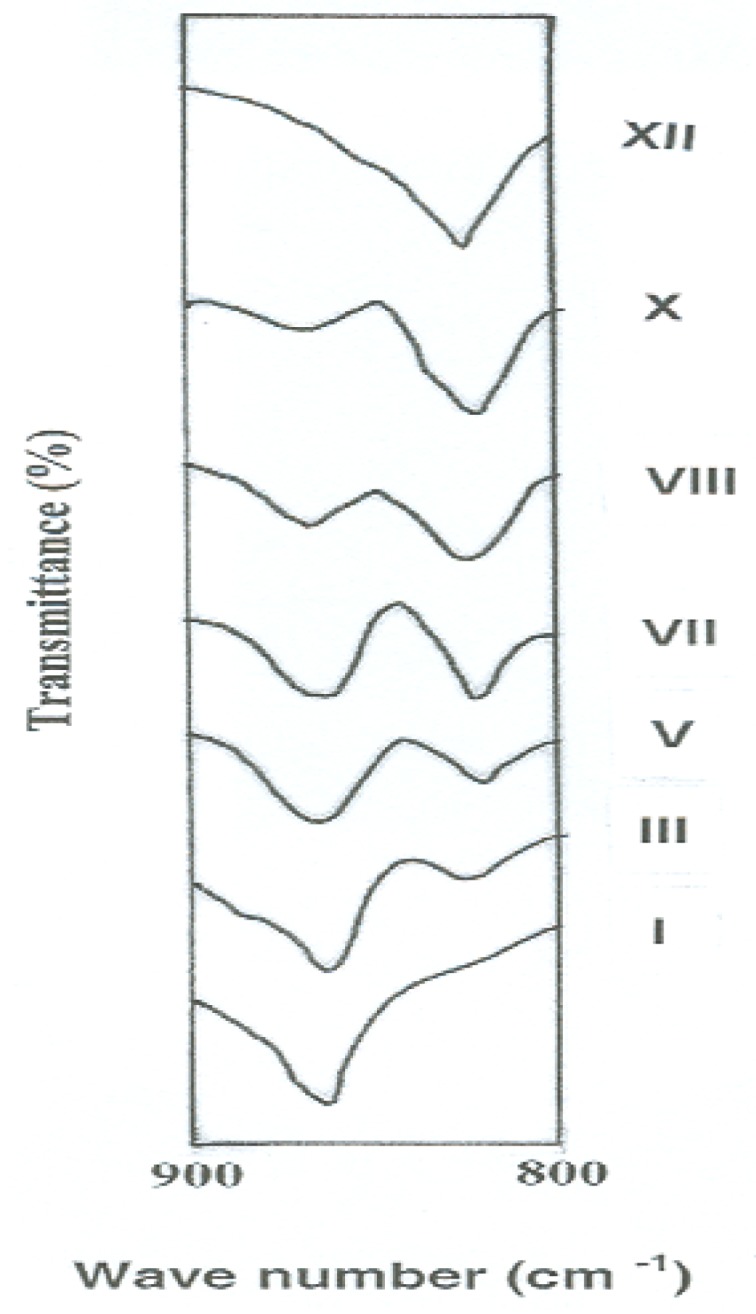
Changes of FTIR peak intensities of novel wholly aromatic azopolyamide-hydrazides as a function of their *para*-phenylene and *meta*-phenylene rings content.

Specific assignment of the band due to -N=N- stretching was not possible because of the overlap by the band corresponding to C=C stretching in aromatic rings [35,36]. Further, it would be expected that all of these polymers have the same structural formula except for the mode of linking of phenylene rings in the polymer chains. This expectation was evidenced experimentally by comparing the FTIR spectra of the polymers in the region of 900–800 cm^−1^ which is characteristic for aromatic C-H out-of-plane bending vibration. In this experiment, thin polymer films having similar thickness of 3 μm were tested in order to obtain a fair detection of any change in the spectra. Figure 1 illustrates the variations of the intensities of FTIR peaks of these polymers in the aforementioned region as a function of their *meta*- to *para*-phenylene moieties content. It can be noted that the *meta*- to *para*-phenylene unit compositions of the polymers changed quantitatively in proportion to their compositions in feed. FTIR spectra showed a gradual increase in the intensity of the band corresponding to the *meta*-phenylene moiety (at 825 cm^−1^), together with a reduction of that of the *para*-phenylene ring (at 865 cm^−1^) from polymer **I** to **XII**.

**Figure 2:**
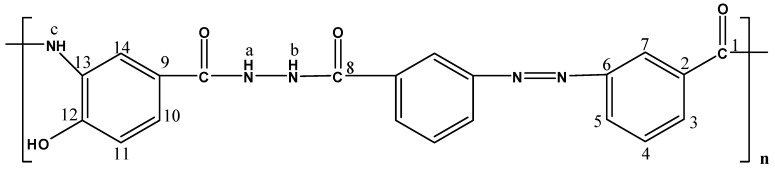
Polymer **XII**.

**Figure 3:**
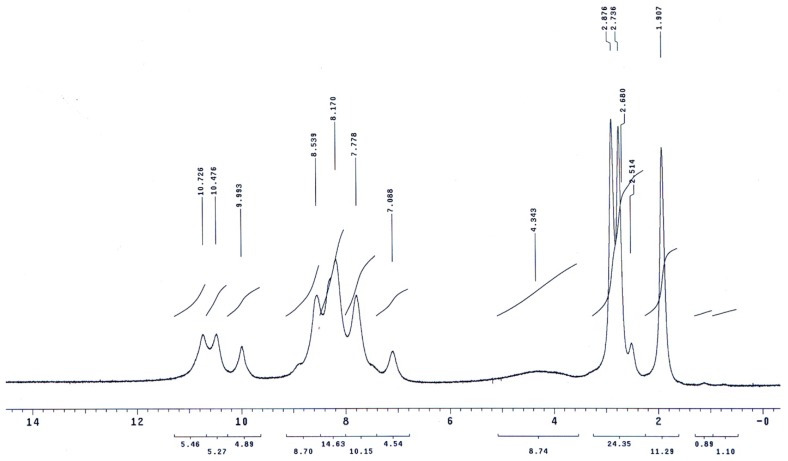
^1^H-NMR spectrum of polymer **XII** in DMSO-*d*_6_.

**Figure 4:**
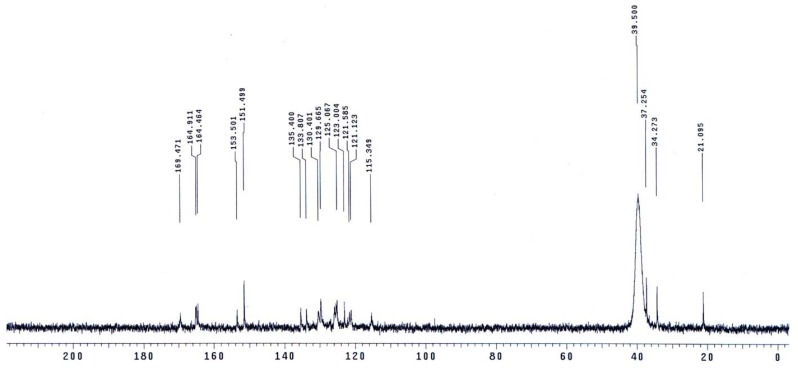
^13^C-NMR spectrum of polymer **XII** in DMSO-*d*_6_.

The molecular structures of the evaluated polymers were also confirmed by NMR analysis. The ^1^H-NMR and ^13^C-NMR spectra of polymer **XII** (Figure 2), as an example, are shown in Figure 3 and Figure 4, respectively. In the ^1^H-NMR spectrum (200 MHz, DMSO-*d_6_*), the polymer showed the following characteristic peaks: δ = 10.73 (s, 1H, OH, D_2_O exchangeable), 10.47 (s, 1H, NH_b_, D_2_O exchangeable), 10.01 (s, 1H, NH_a_, D_2_O exchangeable), 7.08–8.59 ppm (m, 13H, 12 Ar. H+NH_c_). In the ^13^C-NMR (300 MHz, DMSO-*d_6_*) spectrum, the characteristic resonances of the carbon atoms are as follows: δ = 169.4 (C^1^=O), 164.9 (C^8^=O), 153.5 (C^6^), 151.5 (C^12^), 135.4 (C^2^), 133.8 (C^3^), 130.4 (C^4^), 129.6 (C^13^), 125.1 (C^9^), 123.0 (C^5^), 121.6 (C^10^), 121.1 (C^7^), 115.1 (C^14^), 113.0 ppm (C^11^).

The results of elemental analyses and FTIR spectra coupled with the results of ^1^H-NMR and ^13^C-NMR spectra seemed to be in good agreement with the expected structures of the prepared polymers as illustrated in Scheme 1 and Scheme 2.

### 2.3. Polymer Characterization

#### 2.3.1. Intrinsic Viscosity

Table 1 lists the intrinsic viscosity values for the prepared azopolyamide-hydrazides. These values reflect the high molecular weight of the polymers. It can be noted that the intrinsic viscosity of these polymers [measured in DMAc/3% (wt/v) LiCl at 30 °C with a concentration of 0.5 g dL^−1^] increases by increasing the *para*-oriented phenylene rings content and ranges between 0.71 and 4.75 dL g^−1^ when the *para*-phenylene rings content increases from 0 to 100 mol %. This indicates that the polymers’ chain rigidity increases as a function of their *para*-phenylene linkages. The polymer with all *meta*-linkages has the lowest intrinsic viscosity, whereas that with all *para*-linkages has the highest one. The polymers with various proportions of *meta*- to *para*-linkages have intermediate intrinsic viscosities in between these two extreme values. Although the intrinsic viscosity values are generally higher for polymers having high *para*-phenylene rings content, it is expected that the molecular weight of these polymers are generally comparable since the purity level of the two acid chlorides used are similar. The higher intrinsic viscosity value of wholly *para*-oriented type of polymer can be attributed to the higher rigidity, large interchain hydrogen-bonding, associated with higher chain symmetry and packing efficiency. This observation seems to support the results which were previously reported [20,37,38].

#### 2.3.2. Solubility

All the evaluated polymers are found to be partially soluble in polar aprotic solvents like DMAc, DMF and NMP at room temperature. Addition of lithium chloride enhances their solubility. The maximum solubility (%wt/v) of the polymers at 25 °C in these solvents containing various amounts of lithium chloride is given in Table 3. It can be noted from this table that polymer with all *para*-linkages appears to be less soluble relative to other polymers containing different proportions of *meta*-linkages and forms high viscous solutions even at lower concentrations; however, at higher concentrations these solutions appear as gels. Generally, the solubility characteristic of the polymers is in the order: **XII** > **XI** > **X** > **IX** > **VIII** > **VII** > **VI** > **V** > **IV** > **III** > **II**> **I**.

An increase in *meta*-phenylene moieties content of the polymers from 0 to 100 mol % resulted in an increase in its solubility from 10 to 28 wt/v % in DMAc/5% (wt/v) LiCl. This behavior provides additional evidence for the increased rigidity, packing efficiency and hydrogen bonding of wholly *para*-oriented type of polymer. This observation would seem to be supported by the conclusion made earlier by Satre *et al*. [38].

#### 2.3.3. Percent Moisture Regain

All the prepared azopolyamide-hydrazides showed the percent moisture regain (%MR) between 18.67% and 26.29% (at 23 °C, relative humidity, RH 85%) as represented in Table 3. This high moisture uptake reflects the hydrophilic/polar character of these polymers. The presence of a hydrazide group, a hydroxyl group, an amide group in addition to an azo group enhance the moisture uptake relative to Nomex [poly(*meta*-phenylene isophthalamide)] (12% MR, at 25 °C, RH 85%) [39]. In the Nomex repeating unit, there is one amide group compared to the aforementioned four functional groups of the evaluated polymers. As most of the polymers’ polarity is due to functional groups, the azopolyamide-hydrazides are more polar and hence attract larger water sorption. Further, the hydrophilicity is increased with increasing the meta-oriented phenylene rings content in the investigated polymer. This observation may be explained by the wholly *para*-oriented type polymer I being more strongly hydrogen bonded than those containing *meta*-phenylene rings as a result of its greater chain symmetry and shorter interchain distance, while the separation between the macromolecular segments of *meta*-phenylene containing polymers would be further expected, particularly in the presence of water, and consequently the inter- and intra-hydrogen bonds would be partially sterically prevented. Thus they are free to interact with water.

#### 2.3.4. X-ray Diffraction

**Figure 5:**
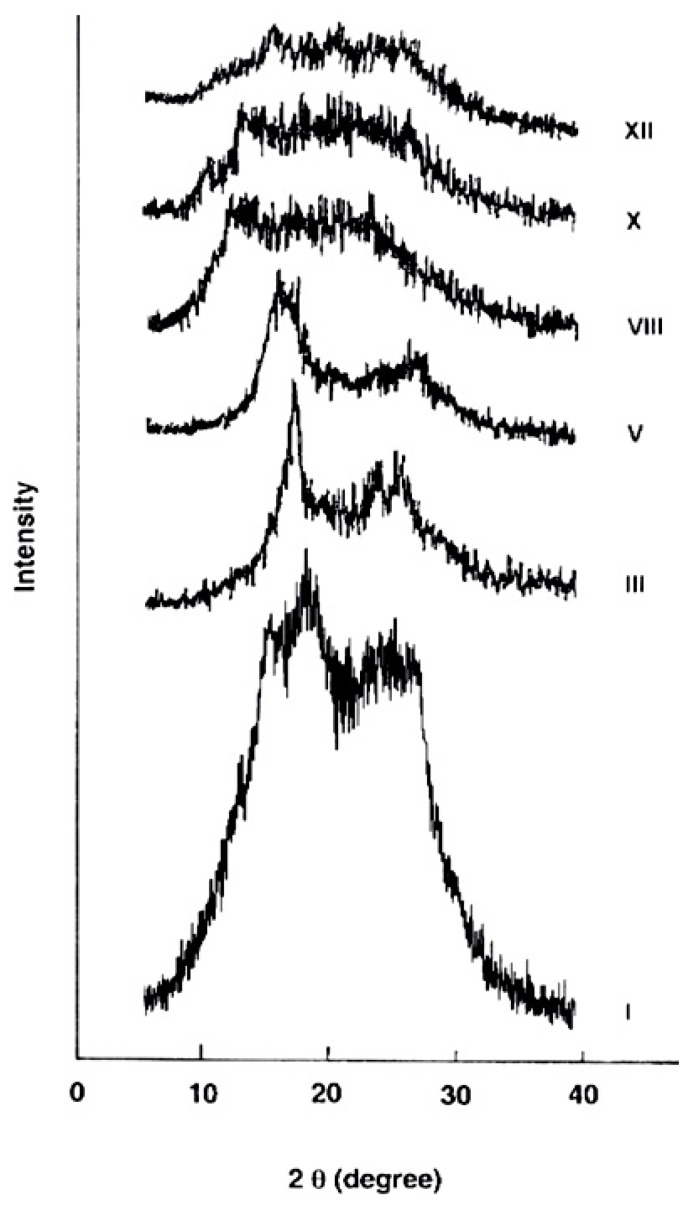
Wide-angle X-ray diffractograms of the novel wholly aromatic azopolyamide-hyrdrazides containing various proportions of *para*-phenylene and *meta*-phenylene rings.

The wide-angle X-ray diffraction patterns of the evaluated polymers over the 2θ range of 5–40° are shown in Figure 5. The polymer **I** derived from 4A3HBH and 4,4′ADBC (completely *para*-oriented type polymer) revealed a higher level of crystallinity consistent with its lower solubility and indicating more order structure and efficient packing. At the molecular level, it is expected that an increase in the polymer chain rigidity is associated with a higher *para*–phenylene rings content which develops denser packing of the macromolecular segements. This dense packed polymer segements are expected to be held together by short-range intermolecular hydrogen bonds. Gradual changing of the 4A3HBH moiety to 3A4HBH and 4,4′ADBC to 3,3′ADBC (polymers **III**, **V**, **VIII**, and **X**) reduced the polymer crystallinity. The polymer **XII** derived from 3A4HBH and 3,3′ADBC (completely *meta*-oriented type polymer) reveals a nearly amorphous pattern. This may be attributed to the fact that the regularity of the repeat units in polymer **XII** is disrupted by the *meta*-oriented phenylene rings, thus leading to a lower crystallinity.

#### 2.3.5. Mechanical Properties

Flexible and tough films were obtained in all cases by evaporation of solvent (DMAc/3% (wt/v) LiCl) from the cast polymers solutions. Mechanical properties of these films before and after being drawn at various temperatures are shown in Table 4. The unoriented films showed good mechanical properties. In general, elastic modulus as well as tensile strength are increased as a function of *para*-phenylene units content in the tested polymer. An increase in *para*-phenylene rings content from 0 to 100 mol % resulted in an increase in the elastic modulus and tensile strength from 0.74 to 5.81 GPa and from 23.42 to 287.56 MPa, respectively. On the other hand, ductility of these polymers is increased by increasing the *meta*-phenylene units content in the prepared polymer as displayed by the values of elongation-to-break. Several attempts were made to improve the mechanical properties of these films by heat drawn as shown in Table 4, but their success was limited.

#### 2.3.6. Thermal Properties

**Figure 6:**
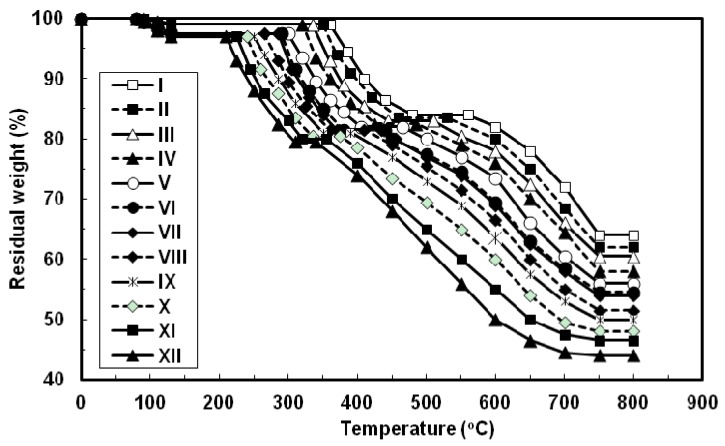
Typical TG thermograms patterns of novel wholly aromatic azopolyamide-hydrazides containing various proportions of *para*-phenylene and *meta*-phenylene rings. All the thermograms were recorded in nitrogen atmosphere at a heating rate of 10 °C min^−1^ and under a gas flow rate of 30 mL min^−1^.

**Figure 7:**
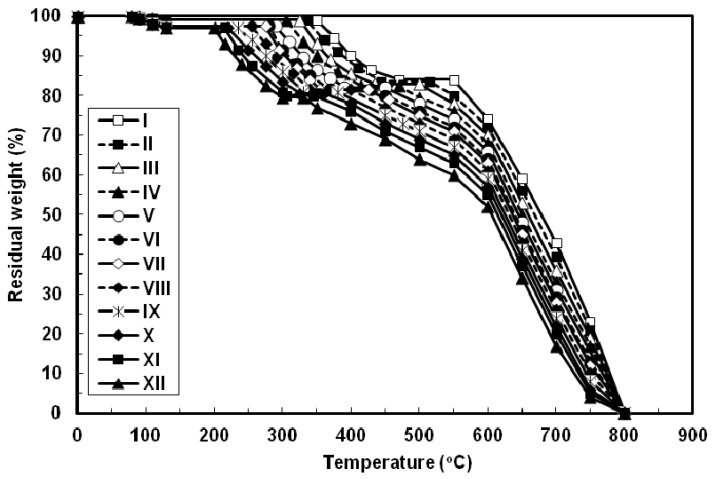
Typical TG thermograms patterns of novel wholly aromatic azopolyamide-hydrazides containing various proportions of *para*-phenylene and *meta*-phenylene rings. All the thermograms were recorded in air atmosphere at a heating rate of 10 °C min^−1^ and under a gas flow rate of 30 mL min^−1^.

The thermal properties of all the azopoly(amide-hydrazide)s were evaluated by thermogravimetry (TG) in nitrogen and in air atmospheres at a heating rate of 10 °C min^−1^ and the typical TG curves thus obtained are shown in Figure 6 and Figure 7, respectively. In both degradation atmospheres, all the polymers showed characteristic similar thermal behavior and their weight losses could be observed in three distinctive steps. First, a small weight loss in the range of 1–3 wt% (based on the original polymer weight) occurred starting at 80 °C up to 130 °C, which may be attributed to loss of absorbed moisture. Second, a considerable weight loss (15–17.5 wt%, based on the weight of the perfectly dried polymer) was observed at various temperature ranges for different polymers in nitrogen and in air atmospheres. This weight loss is attributed to loss of water during the thermally induced conversion of the hydrazide and *o*-hydroxybenzamide groups to 1,3,4-oxadiazole and benzoxazole rings, respectively [12,23,40]. Along with this cyclodehydration reaction, the polymers may lose the azo group as molecular nitrogen [41,42]. Third, a sharp weight loss was obtained which indicated the decomposition of the poly(1,3,4-oxadiazolyl-benzoxazole)s which were formed *in situ*. The almost identical nature of the curves in both atmospheres indicates that the decompositions occur by the same mechanism. The improved resistance to high temperature was always associated with increased *para*-phenylene rings content of the evaluated polymers. In both degradation atmospheres and at all applied temperatures, polymer **I** showed the highest thermal stability as judged by its highest initial decomposition temperatures as well as its highest residual weight at a particular temperature, whereas the reverse was true for polymer **XII**. The other polymers of this series aligned themselves in between these two extreme cases so that with respect to their weight remaining at any particular temperature their order of stability was: **I** > **II** > **III** > **IV** > **V** > **VI** > **VII** > **VIII** > **IX** > **X** > **XI** > **XII**. Thus, substitution of *para*-phenylene nuclei for *meta*-phenylene improved the thermal stability of the polymer. This should also be associated with regularity of supermolecular packing within the bulk of the evaluated polymers wherein the collinear arrangement of the *para*-phenylene units should allow for establishment of stronger intermolecular hydrogen bonds which would be more difficult to break and therefore more resistance to elevated temperature. The resulting poly(1,3,4-oxadiazolyl-benzoxazole)s start to degrade in the temperature range above 340–560 °C in nitrogen and 330–550 °C in air without weight loss at a lower temperature. The percentage residues of poly(1,3,4-oxadiazolyl-benzoxazole)s at 750 °C in nitrogen were 64.5–80% of their original weights. On the other hand, smaller residues of 24.5–39% were observed at the same temperature in air. This indicates that the degradation rate of poly(1,3,4-oxadiazolyl-benzoxazole)s in air is faster than in nitrogen supported by the residual weight of the polymer at 750 °C as well as at particular temperatures. These observations confirm that azopoly(amide-hydrazide)s can be used as precursor polymers for the preparation of thermally stable poly(1,3,4-oxadiazolyl-benzoxazole)s. The detailed thermal and thermo-oxidative characterization of these polymers is in progress.

#### 2.3.7. Ultraviolet-Visible Spectroscopy

**Figure 8:**
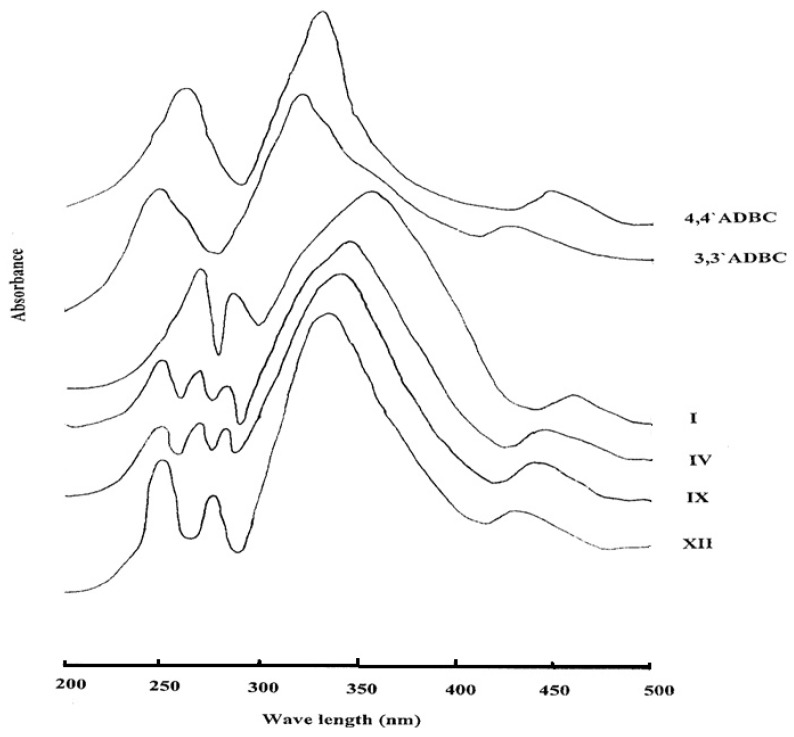
The ultraviolet-visible absorption spectra of some novel wholly aromatic azo polyamide-hydrazides containing various proportions of *para*-phenylene and *meta*-phenylene units.

All the azopolyamide-hydrazides display orange colorations of varying intensity owing to the presence of the azo groups. The ultraviolet-visible absorption spectra of selected examples of azopolyamide-hydrazides **I**, **IV**, **IX**, and **XII**, which were measured in NMP solution (3 × 10^−4^ g/mL) are shown in Figure 8. It was found that the shape of the absorptions of all the investigated polymers was almost the same. The spectra showed a broad and intensive absorption between 300 and 400 nm corresponding to the π-π* transition of the *trans* form of the azo group present in the polymer main chain. A weak and broad absorption between 400 and 500 nm due to the n-π* electronic transition of the *cis* form of the azo group was also observed. The spectra showed absorption maxima at 355 and 460 nm for polymer I, at 345 and 445 nm for polymer IV, at 340 and 440 nm for polymer IX and 335 and 430 nm for polymer XII. It can be noted that the absorption maxima of the polymers were shifted toward a longer wave length with increasing their *para*-oriented phenylene units content. This may be attributed to extension of the conjugation through the azo group in the all-*para*-linked polymer relative to the other polymers. Moreover, all azopolyamide-hydrazides exhibited some peaks in the ultraviolet region which are the benzenoid bands for the various types of benzene rings derived from either 4A3HBH (at 284 ± 1 nm) or 3A4HBH (at 278 ± 2 nm) and 4,4′ADBC (at 270 nm) and/or 3,3′ADBC (at 250 nm). The spectrum of 4,4′ADBC shows absorption maxima at 445 (n-π*, azo), 330 (π-π*, azo) and 260 nm (benzenoid band), whereas, the spectrum of 3,3′ADBC shows absorption maxima at 425 (n-π*, azo), 320 (π-π*, azo) and 250 nm (benzenoid band). The red shift for the π-π* transition in the polymers is due to the extension of the conjugation of the azo group by the hydrazide group in the polymer main chain.

The spectra of 4A3HBH and 3A4HBH showed benzenoid bands at 300 and 290 nm, respectively. The blue shift of these bands in the polymers is due to less availability of the lone pair of electrons on hydrazide nitrogen for conjugation with the benzene ring as it also can undergo conjugation with the carbonyl group.

## 3. Experimental

### 3.1. Synthesis of the Monomers

4-Amino-3-hydroxybenzhydrazide (4A3HBH) and 3-amino-4-hydroxy-benzhydrazide (3A4HBH) were synthesized from the corresponding acids by two-step procedure in which the acid was esterified to its methyl ester followed by reaction of the produced ester with hydrazine hydrate. A detailed description of this procedure is given in our previous work [43].

**Figure 9:**
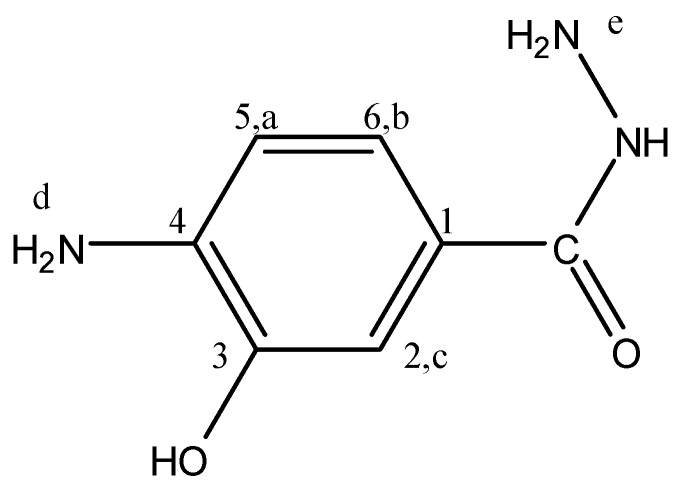
4A3HBH.

*4-Amino-3-hydroxybenzhydrazide* (4A3HBH, Figure 9) separated as felted needles (45% yield) m.p. 223–224 °C; FTIR: ν_max_ 3460 (OH str.), 3356, 3252 (N-H str.), 3022 (C-H str.), 1667 (C=O str.), 1604 cm^−1^ (Ar. C=C str.); ^1^H-NMR (200 MHz, DMSO-*d_6_*): δ = 9.25 (s, 1H, OH), 7.19 (s, 1H, H_c_), 7.13 (d, 1H, H_b_, *J* = 7.6 Hz), 6.56 (d, 1H, H_a_, *J* = 7.6 Hz), 5.00 (s, 1H, NH), 4.33 (s, br., 2H, NH_2,d_), 3.49 ppm (s, br., 2H, NH_2,e_); ^13^C-NMR (300 MHz, DMSO-*d_6_*): δ = 166.77 (C=O), 143.06 (C^3^), 140.11 (C^4^), 120.9 (C^1^), 119.10 (C^6^), 113.62 (C^5^), 112.76 ppm (C^2^); MS m/z (%): 167 (9.7, M^+^), 136 (100.0), 108 (14.2), 80 (42.8), 53 (43.5); Anal. Calcd. for C_7_H_9_N_3_O_2_: C, 50.30%; H, 5.39%; N, 25.15%; O, 19.16%. Found: C, 50.35%; H, 5.4%; N, 25.12%; O, 19.13%.

**Figure 10:**
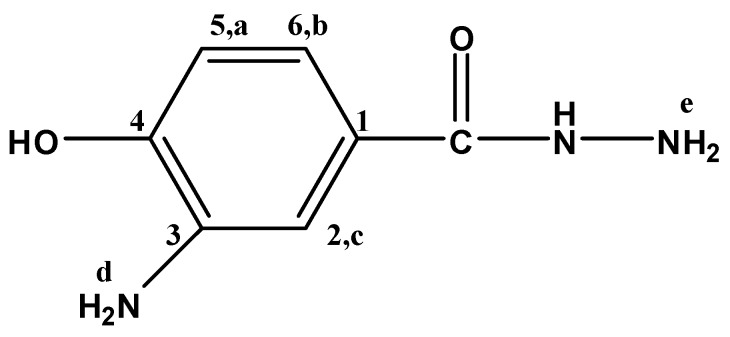
3A4HBH.

*3-Amino-4-hydroxybenzhydrazide* (3A4HBH, Figure 10) separated as felted needles (45% yield) m.p. 227 °C; FTIR: ν_max_ 3390 (OH str.), 3317, 3249 (N-H str.), 3008 (C-H str.), 1662 (C=O str.), 1598 cm^−1^ (Ar. C=C str.); ^1^H-NMR (200 MHz, DMSO-*d_6_*): δ = 9.31 (s, 1H, OH), 7.13 (s, 1H, H_c_), 6.94 (m, 2H, NH, H_b_), 6.64 (d, 1H, H_a_), 4.58 (s, 2H, NH_2,d_), 3.44 ppm (s, 2H, NH_2,e_); ^13^C-NMR (300 MHz, DMSO-*d_6_*): δ = 166.93 (C=O), 146.82 (C^4^), 136.30 (C^3^), 124.76 (C^1^), 115.80 (C^6^), 113.64 (C^5^), 113.55 ppm (C^2^); MS m/z (%): 167 (21.2, M^+^), 136 (100.0), 108 (34.5), 80 (43.2), 53 (39.3); Anal. Calcd. for C_7_H_9_N_3_O_2_: C, 50.30%; H, 5.39%; N, 25.15%; O, 19.16%. Found: C, 50.37%; H, 5.38%; N, 25.09%; O, 19.19%.

**Figure 11:**
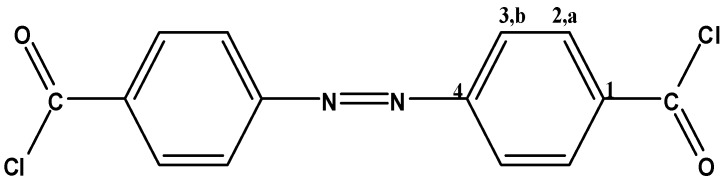
4,4′ADBC.

**Figure 12:**
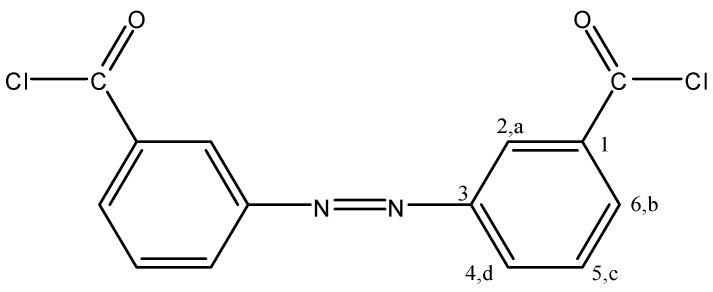
3,3′ADBC.

*4,4′-Azodibenzoyl chloride* (4,4′ADBC, Figure 11) and *3,3′-azodibenzoyl chloride* (3,3′ADBC, Figure 12) were prepared according to the methods previously reported in details [42,44]. 4,4′ADBC separated as red needles (47% yield), mp: 164 °C; FTIR: ν_max_ 2925 (C-H str.), 1776 (C=O str.), 1595 (Ar. C=C str.), 1151, 885 cm^−1^ (C-Cl); ^1^H-NMR (200 MHz, DMSO-*d_6_*): δ = 8.18 (d, 4H, H_a_, *J* = 8.6 Hz), 8.00 ppm (d, 4H, H_b_, *J* = 8.6 Hz); ^13^C-NMR (300 MHz, DMSO-*d_6_*): δ = 166.60 (C=O), 154.25 (C^1^), 133.43 (C^4^), 130.74 (C^2^), 122.86 ppm (C^3^); MS m/z (%): 306 (12.08, M^+^−1), 271 (25.09), 167 (24.03), 139 (55.59), 104 (65.44), 76 (100.00), 50 (49.77); Anal. Calcd. for C_14_H_8_N_2_Cl_2_O_2_: C, 54.72%; H, 2.62%; N, 9.12%; Cl, 23.09%; O, 10.45%. Found: C, 54.69%; H, 2.64%; N, 9.09%; Cl, 23.11%; O, 10.47%.

3,3′ADBC separated as orange crystals (51% yield), mp: 97–98 °C; FTIR: ν_max_ 2925 (C-H str.), 1776 (C=O str.), 1595 (Ar.C=C str.), 1151, 885 cm^−1^ (C-Cl); ^1^H-NMR (200 MHz, DMSO-*d_6_*): δ = 8.4 (s, 2H, H_a_), 8.12–8.24 (m, 4H, H_b_, H_d_), 7.71–7.83 ppm (m, 2H, H_c_); ^13^C-NMR (300 MHz, DMSO-*d_6_*): δ = 166.60 (C=O), 151.80 (C^1^), 132.28 (C^6^), 130.03 (C^3^), 129.53 (C^4^), 127.53 (C^5^), 122.49 ppm (C^2^); MS m/z (%): 306 (18.76, M^+^-1), 271 (39.29), 167 (45.13), 139 (100.00), 103 (45.01), 76 (78.97), 50 (27.57); Anal. Calcd. for C_14_H_8_N_2_Cl_2_O_2_: C, 54.72%; H, 2.62%; N, 9.12%; Cl, 23.09%; O, 10.45%. Found: C, 54.75%; H, 2.63%; N, 9.04%; Cl, 23.08%; O, 10.50%.

### 3.2. Synthesis of the Polymers

All the polymers were prepared by essentially the same experimental procedure, which will be given here for preparation of polymer **I**. A dry 1 L four-necked round bottom flask equipped with a Teflon coated halfmoon mechanical stirring bar, a thermometer and a drying tube was placed in a dry box. 4A3HBH, 16.7 g (0.1 mol), was charged into the reaction flask followed by 400 mL of anhydrous DMAc containing 3% (wt/v) LiCl. Stirring was started until complete dissolution. The reaction flask was placed in a crushed ice- salt water bath and cooled at −10 °C for 15 min. When this step was completed, 30.7 g (0.1 mol) solid 4,4′ADBC was added slowly under constant stirring over a period of 1 h by means of a funnel with weighing paper that was positioned just slightly above the surface of the stirred polycondensation reaction solution. After this period, stirring was continued for another 2 h at the aforementioned temperature. Then the cooling bath was removed and the temperature of the polymerization reaction was allowed to rise gradually to room temperature and maintained for 24 h with stirring. Afterwards, a clear, orange, highly viscous solution was obtained. Finally, the polymer solution was slowly poured into 3 L of rapidly stirred methanol upon which a fibrous orange precipitate of polymer I immediately formed. The polymer was isolated by filtration, washed successively with methanol, acetone and dried in a vacuum oven at room temperature to constant weight.

### 3.3. Film Preparation

Films were prepared by casting 5% (wt/v) polymer solutions (in DMAc containing 3% (wt/v) anhydrous LiCl) onto dry clean Pyrex glass plates to a uniform thickness. Solvent evaporation was performed in a vacuum oven at room temperature. The resulting films were kept in the oven until no change in weight could be observed. The films were then immersed in deionized water overnight to remove any residual solvent. Finally, the films were dried in a vacuum oven at room temperature to constant weight. The thickness of the films was 0.3 ± 0.02 mm.

### 3.4. Measurements and Instruments

Fourier transform infrared spectra were recorded on a Perkin-Elmer infrared spectrophotometer (FTIR 1650) using KBr pellets. All spectra were recorded within the wave number range of 4000–400 cm^−1^ at 25 °C. ^1^H- and ^13^C-NMR spectra were recorded on a 200 MHz and 300 MHz Varian Gemini NMR spectrometer, respectively. Deuterated dimethyl sulphoxide (DMSO-*d_6_*) was used as the solvent in all cases at 25 °C. Tetramethylsilane (TMS) was used as an internal standard. Mass spectra were recorded under electron impact at 70 eV on a GCMS Analysis Shimadzu GP 2010 Plus Japan instrument. Elemental analyses were performed at the Microanalytical Unit, Cairo University, Giza, Egypt. Intrinsic viscosity measurements were determined for all of the polymers in DMAc/3% (wt/v) LiCl at 30 °C. Solutions were prepared at room temperature in all cases. Their concentration was 0.5 g dL^−1^. Flow times were determined for each polymer at five different concentrations using a cannon viscometer which was selected so as to give solvent flow times in excess of 100 seconds. All flow times encountered were sufficiently long to justify neglect of kinetic energy corrections, and the lack of shear- rate dependence was verified. The plots obtained were linear in all cases. Intrinsic viscosities were determined by the usual double extrapolation of η_sp_/C and (ln η_rel_)/C to zero concentration and expressed in deciliter per gram (dL g^−1^).

The solubility of polymers in various amide solvents namely DMAc, DMF, and NMP, containing various amounts of LiCl, was determined at room temperature (25 °C). The saturated solutions of the polymers in solvent were prepared at room temperature by equilibrating for 24 h. The solubilities of the polymers were recorded as percent weight dissolution per hundred mL of solvent (%wt/v).

A dried powder polymer sample was allowed to absorb moisture for 24 h in a humidity chamber maintained at 85% relative humidity (RH) at 23 °C, then a weighed amount of the moisture-absorbed sample was dried in an air oven at 110 °C to constant weight. The percent moisture regain by the polymer sample was calculated on the basis of weight loss.

Wide-angle X-ray diffraction measurements were performed at room temperature on a Rigaku Denki Co. Ltd. (Tokyo, Japan), RAD-B system X-ray diffractometer, using Ni filtered Cuk_α_ radiation (operating at 40 kV and 20 mA). The sample was maintained stationary, while scattering angles from 5 to 90° were scanned in the reflection mode at scanning rate of 1° min^−1^.

Tensile strength (T), elastic modulus (M) and elongation-to-break (%) (E) of the polymers films were measured on Shimadzu Autograph in air at room temperature.

Thermogravimetric measurements of all the polymers were carried out in nitrogen and in air atmospheres. A Shimadzu TGA-50H thermogravimetric analyzer was used. The samples ranging from 4 to 6 mg in weight were heated from 25 to 800 °C at a heating rate of 10 °C min^−1^ and under a gas flow stream of 30 mL min^−1^ in all cases. Ultraviolet-visible absorption spectra of the prepared azopolymers were recorded in NMP solution (3 × 10^−4^ g/mL) using a Shimadzu UV-1600 series spectrophotometer in the range 200–800 nm.

## 4. Conclusions

High molecular weight easily processed novel self-dyed wholly aromatic azopolyamide-hydrazides have successfully been synthesized as highly viscous film forming solutions. They show orange colors of varying intensity owing to the presence of the azo groups. They were structurally characterized by various *para*- to *meta*-phenylene moieties contents. These polymers exhibited some desirable properties which mark them as promising candidates for corresponding engineering applications and perhaps also for some new ones. Substitution of *para*-phenylene units for *meta*-phenylene ones within these polymers greatly affected their physical, mechanical and thermal properties. The intrinsic viscosity, tensile strength, crystallinity, thermal and thermo-oxidative stability of the polymers increased as a function of *para*-phenylene units content in the polymer. These properties indicate the regularity of supermolecular packing occurred with the bulk of the polymer in addition to the collinear arrangement of *para*-phenylene units which permit the establishment of stronger inter-chain hydrogen bonding. The latter would also increase the rigidity of the polymer. On the other hand, the solubility and the hydrophilic character of the polymers increased as a function of *meta*-oriented phenylene rings content incorporated into the polymer chains. The gradual substitution of *para*-phenylene rings in the chains of the polymer by the more flexibilizing 4*meta*-phenylene units gradually increases the overall flexibility of the resulting polymer chains. Thus, it should become possible to prepare materials with the most favorable properties required for any given engineering application or desired processing conditions by carefully designing relative ratios of the two types of these aromatic units during the polymer synthesis.

## Figures and Tables

**Table 1:** Syntheses of novel wholly aromatic azopolyamide-hydrazides containing various proportions of para-phenylene and meta phenylene units.

Polymer code	Monomer feed composition (mol %)				Expected *para/meta*-phenylene rings in polymer (mol %)	Intrinsic viscosity ^a^ (dL g^−1^)	Yield (%)
	4A3HBH	3A4HBH	4,4′ADBC	3,3′ADBC			
**I**	100	-	100	-	100/0	4.75	99.85
**II**	100	-	80	20	90/10	3.98	99.64
**III**	100	-	60	40	80/20	3.51	99.46
**IV**	100	-	40	60	70/30	2.83	99.38
**V**	100	-	20	80	60/40	2.64	99.81
**VI**	100	-	-	100	50/50	2.26	99.80
**VII**	-	100	100	-	50/50	2.21	99.77
**VIII**	-	100	80	20	40/60	1.80	99.45
**IX**	-	100	60	40	30/70	1.47	99.57
**X**	-	100	40	60	20/80	1.22	99.83
**XI**	-	100	20	80	10/90	1.08	99.95
**XII**	-	100	-	100	0/100	0.71	99.78

^a^ Determined in DMAc/3%(wt/v) LiCl at 30 °C, polymer concentration was 0.5 g dL^−1^.

**Table 2:** Elemental analysis data of novel wholly aromatic azopolyamide-hydrazides containing various proportions of *para*-phenylene and *meta*-phenylene units.

Polymer code	Elemental analyses			
	% C	% H	% N	% O ^a^
Calculated for the expected repeating unit, C_21_H_15_N_5_O_4_	62.84	3.74	17.46	15.96
**I**	62.83	3.76	17.41	16.00
**II**	62.79	3.75	17.43	16.03
**III**	62.81	3.78	17.40	16.01
**IV**	62.78	3.76	17.42	16.04
**V**	62.76	3.79	17.39	16.06
**VI**	62.72	3.77	17.44	16.07
**VII**	62.80	3.80	17.38	16.02
**VIII**	62.73	3.80	17.40	16.07
**IX**	62.75	3.79	17.37	16.09
**X**	62.70	3.81	17.34	16.15
**XI**	62.74	3.81	17.33	16.12
**XII**	62.71	3.82	17.36	16.11

^a^ Calculated as % O = 100 − (% C + % H + % N).

**Table 3:** Maximum solubility and percent moisture regain of the novel wholly aromatic azopolyamide-hydrazides containing various proportions of *para*-phenylene and *meta*-phenylene units.

Polymer code	Maximum solubility (%wt/v)									Moisture regain (%) ^a^
	DMAc/LiCl(%wt/v)			DMF/LiCl(%wt/v)			NMP/LiCl(%wt/v)			
	1	3	5	1	3	5	1	3	5	
**I**	2	5	10	1	3	6	1	3	7	18.67
**II**	2	6	12	2	3	7	2	3	9	19.11
**III**	3	6	14	2	4	8	3	5	9	20.22
**IV**	5	9	17	3	6	11	5	7	13	21.78
**V**	6	10	21	4	7	12	6	8	15	22.48
**VI**	8	13	22	6	10	14	7	11	18	23.58
**VII**	9	13	22	7	9	14	6	12	19	23.83
**VIII**	10	15	24	8	11	17	9	13	21	24.52
**IX**	11	16	25	10	12	19	11	15	24	24.93
**X**	13	17	25	12	14	23	14	17	24	25.28
**XI**	15	18	26	14	17	23	17	20	27	25.93
**XII**	15	20	28	14	19	25	19	22	29	26.29

^a^ Determined at 23 °C and relative humidity 85%.

**Table 4:** Mechanical properties of novel wholly aromatic azopolyamide-hydrazides containing various proportions of para-phenylene and meta-phenylene units.

Polymer code	Mechanical properties ^a^											
	As cast			Heat drawn ^b^								
				At 140 °C			At 160 °C			At 180 °C		
	M	E	T	M	E	T	M	E	T	M	E	T
**I**	5.8	47.3	287.6	9.3	26.3	378.1	14.9	16.3	477.9	30.9	6.2	885.6
**II**	4.8	68.5	249.6	7.9	34.2	326.9	12.8	19.8	408.7	26.1	9.3	739.2
**III**	3.9	91.8	213.3	6.5	42.1	271.4	10.5	23.8	336.2	20.1	12.3	595.3
**IV**	3.5	100.2	181.4	5.4	51.0	220.6	9.1	29.1	215.4	17.6	13.5	555.6
**V**	2.8	108.2	147.2	4.2	60.2	168.8	7.6	34.7	191.7	15.0	14.5	515.9
**VI**	2.5	121.4	116.7	4.1	63.9	148.6	6.7	36.6	179.1	13.2	17.3	449.6
**VII**	2.0	136.4	87.8	3.9	68.6	126.9	5.5	38.9	166.1	11.2	20.9	383.5
**VIII**	1.8	147.2	62.3	3.2	80.1	118.2	4.2	42.6	155.9	10.1	22.5	312.3
**IX**	1.5	157.9	36.5	2.3	92.5	108.1	3.7	46.6	144.4	8.9	24.7	237.6
**X**	1.3	167.7	31.1	1.9	100.2	91.7	3.0	53.7	120.9	7.4	29.4	198.1
**XI**	1.1	177.5	26.1	1.6	108.5	72.4	2.4	60.7	98.1	5.8	32.9	158.4
**XII**	0.7	181.2	23.4	1.1	120.3	56.6	1.6	67.9	74.7	4.4	35.2	138.6

^a^ Measured at room temperature, M is the elastic Yong’s modulus (GPa), T is the tensile strength (MPa), and E is the elongation-at-break (%); ^b^ Achieved by hand at a preselected temperature till break (maximum stretching).

## References

[1] Zhu Q.M., Dong L.J., Niu Y.S., Xiong C.X., Liu Y., Liu J.T., Liu L.P., Shu B.Q. (2008). Synthesis and characterization of novel fluorinated azopolyamides. Polym. Bull..

[2] Blair H.S., Pague H.I., Riordan J.E. (1980). Photoresponsive effects in azo polymers. Polymer.

[3] Rajendran V., Nanjan M.J. (1989). Thermal behavior and fiber studies of certain azoaromatic polyamides. J. Appl. Polym. Sci..

[4] Morgan P.W. (1974). Polyamide spinning dope. U.S. Patent.

[5] Ho C.H., Yang K.N., Lee S.N. (2001). Mechanistic study of trans-cis isomerism of the substituted azobenzene moiety bounded on a liquid-crystalline polymer. J. Polym. Sci. A.

[6] Cui L., Tong X., Yan X., Liu G., Zhao Y. (2004). Photoactive thermoplastic elastomers of azobenzene-containing triblock copolymers prepared through atom transfer radical polymerization. Macromolecules.

[7] Kim T.D., Lee G.U., Lee K.S., Kim O.K. (2000). Synthesis and characterization of a novel polyimide-based second-order nonlinear optical material. Polymer.

[8] Tuo X., Chen Z., Wu L., Wang X., Liu D. (2000). Photoreponsive self-assembled multilayers of three new azo polyelectrolytes. Polym. Prepr..

[9] Meng X., Natansohn A., Barrett C., Rochon P. (1996). Azo polymers for reversible optical storage. Cooperation motion of polar side groups in amorphous polymers. Macromolecules.

[10] He Y., Wang H., Tuo X., Deng W., Wang X. (2004). Synthesis, self-assembly and photoinduced surface- relief grating of a polyacrylate-based azo polyelectrolyte. Opt. Mater..

[11] Abdolmaleki A. (2007). Novel aromatic poly(amide-hydrazide)s based on the bipyridine. Part I: Synthesis, characterization and thermal stability. Polym. Degrad. Stab..

[12] Mohamed N.A., Fahmi M.M. (2009). Synthesis and characterization of novel wholly para-oriented aromatic polyamide-hydrazides containing sulfone-ether linlages. J. Appl. Polym. Sci..

[13] Preston J., Black W.B., Hofferbert W.L. (1973). High modulus wholly aromatic fibers. I. Wholly ordered polyamide-hydrazides and poly-1,3,4-oxadiazole-amides. J. Macromol. Sci. Chem..

[14] Shukla J.S., Misra P.K. (1984). Studies on some newer polyhydrazides containing amide linkages. III. J. Polym. Sci. A Polym. Chem..

[15] Hsiao S.-H., Yu C.-H. (1998). Novel aromatic polyhydrazides and poly(amide-hydrazide)s based on multiring flexible dicarboxylic acids. J. Polym. Sci. A.

[16] Dvornic P.R. (1984). Wholly aromatic polyamide-hydrazides. 2. Rheological properties of poly(4-(terephthaloylamino)benzoic acid hydrazide) in moderately concentrated *N*,*N*-dimethylacetamide solutions. Macromolecules.

[17] Black W.B., Preston J. (1973). High-Modulus Wholly Aromatic Fibers.

[18] Sourirajan S., Matsura T. (1979). Environmental Protection Agency Symposium Proceedings Textile and Technology No. 299132.

[19] McKinney R., Rhodes J.H. (1971). Aromatic polyamide membranes for reverse osmosis separations. Macromolecules.

[20] Dvornic P.R. (1986). Wholly aromatic polyamide-hydrazides. IV. Structure-property relationships for polymers containing *p*-phenylene and *m*-phenylene units. J. Polym. Sci. A.

[21] Mohamed N.A., Al-Dossary A.O.H. (2003). Structure-property relationships for novel wholly aromatic polyamide-hydrazides containing various proportions of para-phenylene and meta-phenylene units. III. Preparation and properties of semi-permeable membranes for water desalination by reverse osmosis separation performance. Eur. Polym. J..

[22] Mohamed N.A. (2007). Structure-property relationships for novel wholly aromatic polyamide-hydrazides containing various proportions of para-phenylene and meta-phenylene units. V. Evaluation of the electrical surface conductivity of several metalized plastic films. Polym. Test..

[23] Dvornic P.R. (1984). Wholly aromatic polyamide-hydrazides. III. Thermal stability and degradation behaviour of poly[4-(terephthaloylamino) benzoic acid hydrazide]. Bull. Soc. Chim. (Beograd).

[24] Mohamed N.A. (1994). Novel wholly aromatic polyamide-hydrazides. V. Structure-molecular-weight-thermal-stability relationships. Polym. Degrad. Stab..

[25] Cassidy P.E. (1980). Thermally Stable Polymers.

[26] Nanjan M.J., Mark H.F., Bikales N.M., Overberger C.G., Menges G., Kroschwitz J.I. (1988). Polyhydrazides and Polyoxadiazoles, In Encyclopedia of Polymer Science and Engineering.

[27] Maglio G., Palumbo R., Tortora M. (2000). Aromatic poly(benzoxazole)s from multiring diacids containing (phenylenedioxy)diphenylene or (naphthalenedioxy)diphenylene groups: Synthesis and thermal properties. J. Polym. Sci..

[28] Moyer W.W., Cole C., Anyos T. (1965). Aromatic polybenzoxazoles. J. Polym. Sci..

[29] Frazer A.H., Sweeny W., Wallenberger F.T. (1964). Poly(1,3,4-oxadiazoles): A new class of polymers by cyclodehydration of polyhydrazides. J. Polym. Sci..

[30] Abshire C.J., Marvel C.S. (1961). Some oxadiazole and triazole polymers. Makromol. Chem..

[31] Morrison R.W., Preston J., Randall J.C., Black W.B. (1973). Self-regulating polycondensation. II. A study of the order present in polyamide-hydrazides derived from terephthaloyl chloride and *p*-aminobenzhydrazide. J. Macromol. Chem..

[32] Randall J.C., Morrison R.W., Preston J. (1973). Self-regulating polycondensation. III. NMR analysis of oligomers derived from terephthaloyl chloride and p-aminobenzhydrazide. J. Macromol. Sci. Chem..

[33] Dvornic P.R. (1983). Wholly aromatic polyamide-hydrazides. I. A viscometrical method for monitoring of condensation polymerization reactions. J. Appl. Polym. Sci..

[34] Panar M., Beste L.F. (1977). Structure of poly(1,4-benzamide) solutions. Macromolecules.

[35] Henniker J.C. (1967). Infra-Red Spectrometry of Industrial Polymers.

[36] Ballamy L.J. (1975). The Infra-Red Spectra of Complex Molecules.

[37] Preston J., Black W.B., Hofferbert W.L. (1973). High modulus wholly aromatic fibers. II. Partially ordered polyamide-hydrazides. J. Macromol. Sci. Chem..

[38] Satre M.D., Ghatge N.D., Ramani M.P.S. (1990). Aromatic polyamide-hydrazides for water desalination. I. Syntheses and RO membrane performance. J. Appl. Polym. Sci..

[39] Strathmann H., Michaels A.S. (1977). Polymer-water interaction and its relation to reverse osmosis desalination efficiency. Desalination.

[40] Culbertson B.M., Murphy R. (1966). Poly (phenylene-1,3,4-oxadiazolyl-benzoxazoles). J. Polym. Sci. C Polym. Lett..

[41] Mandal R., Maiti S. (1997). A new rigid rodlike colored poly(amide-hydrazide): Thermal, optical anisotropic, and mechanical behavior. J. Appl. Polym. Sci..

[42] Riordan J.E., Blair H.S. (1979). Synthesis and characterization of inherently coloured azo polyamides. Polymer.

[43] Mohamed N.A., Al-Dossary A.O.H. (2003). Structure-property relationships for novel wholly aromatic polyamide-hydrazides containing various proportions of para-phenylene and meta-phenylene units. I. Synthesis and characterization. Eur. Polym. J..

[44] Tomlinson M.L. (1946). The reduction of *p*-nitrobenzoic acid to hydrazo- and azo-benzene-4:4′- dicarboxylic acid by means of glucose. J. Chem. Soc..

